# Cheminformatics-based design and biomedical applications of a new Hydroxyphenylcalix[4] resorcinarene as anti-cancer agent

**DOI:** 10.1038/s41598-024-82115-1

**Published:** 2024-12-13

**Authors:** S. F. Alshahateet, R. M. Altarawneh, S. A. Al-Trawneh, Y. M. Al-Saraireh, W. M. Al-Tawarh, K. R. Abuawad, Y. M. Abuhalaweh, M. Zerrouk, A. Ait Mansour, R. Salghi, B. Hammouti, M. Merzouki, R. Sabbahi, L. Rhazi, Mohammed M. Alanazi, K. Azzaoui

**Affiliations:** 1 Department of Chemistry, Faculty of Science, Mutah University, P.O. Box 7, Al-Karak, 61710 Jordan; 2 Department of Pharmacology, Faculty of Medicine, Mutah University, P.O. Box 7, Al-Karak, 61710 Jordan; 3 Engineering Laboratory of Organometallic, Molecular Materials and Environment, Faculty of Sciences, Sidi Mohammed Ben Abdellah University, 30000 Fez, Morocco; 4 Laboratory of Applied Chemistry and Environment, ENSA, University Ibn Zohr, P.O. Box 1136, 80000 Agadir, Morocco; 5 Euromed Research Center, Euromed Polytechnic School, Euromed University of Fes, UEMF, 30030, Fez, Morocco; 6 Laboratory of Applied Chemistry and Environment, Department of Chemistry, Faculty of Sciences, Mohammed 1st University, Oujda, Morocco; 7 Research Team in Science and Technology, Higher School of Technology, Ibn Zohr University, P.O. Box 3007, Laayoune, Morocco; 8 Institut Polytechnique UniLaSalle, Université d’Artois, ULR 7519, 19 Rue Pierre Waguet, BP 30313, 60026 Beauvais, France; 9 Department of Pharmaceutical Chemistry, College of Pharmacy, King Saud University, 11451 Riyadh, Saudi Arabia; 10 Laboratory of Industrial Engineering, Energy and the Environment (LI3E) SUPMTI, Rabat, Morocco

**Keywords:** Anti-cancer agent, Calix[n]arenes, MTT assay, Anti-proliferative activity, Molecular docking, Cancer therapy, Cancer, Computational biology and bioinformatics, Drug discovery

## Abstract

The distinct conformational characteristics, functionality, affordability, low toxicity, and usefulness make calixarene-based compounds a promising treatment option for cancer. The aim of the present study is to synthesize a new calixarene-based compound and assess of its anticancer potential on some human cancer cells. The synthesized C-4-Hydroxyphenylcalix[4] resorcinarene (**HPCR**) was characterized by several spectroscopic techniques such as 1HNMR, 13CNMR, and X-ray crystallographic analysis to confirm its purity and identity. IC_**50**_ values were identified for cancer cell lines (U-87, MCF-7, A549) and human dermal fibroblasts cell line (HDF) after treatment with **HPCR** and the standard drug Cisplatin. A significant selective growth inhibitory activity against U-87 and A549 cell lines was obtained at an **HPCR** concentration of 100 μM. The MOE docking module (version 2015) was utilized to assess the extent of inhibition for **HPCR** compound against four cancer-related proteins (3RJ3, 7AXD, 6DUK, and 1CGL).

## Introduction

Calix[n]arenes are promising basket-shaped macromolecule molecules with anti-microbial, anti-diabetic, anti-microbial, anticancer, anti-obesity and anti-cancer properties^1^. Several studies have reported the development of many Calixarene-based compounds aiming to produce molecules that selectively act on cancers, with no effect seen in normal cells^2–4^. For instance, p-tert-butylcalix[4]arene have been shown to selectively cause growth inhibitory effects against human breast cancer (MCF-7) and human glioblastoma cancer (U-87) cell lines^2^. Another modified Calixarene, containing tetra-amines on its rims, showed anti-proliferation activity against lung carcinoma (A549), breast adeno-carcinoma (MCF-7), and head and neck carcinoma (SQ20B) cell lines^3^. Moreover, several attempts were successful in accommodating the betaine compound to **p-Sulfonatocalix[4]arenes** by guest–host complexation, this complexation showed selective anti-proliferation activity against human breast cancer (MCF-7) cell line, by induction of cancer cells suppressor genes and apoptotic pathways^4,5^.

In contrast, there are many challenges with using Calixarenes as an anti-cancer agent including their low solubility and substituted groups influence. These factors can limit their ability to effectively reach and interact with cells. Additionally, some functionalized Calixarenes can exhibit non-specific interactions with biological molecules, which can lead to unwanted side effects and reduced anti-proliferation effectiveness^1^. Overall, while further research is needed to fully understand the mechanisms behind the anti-cancer activity of Calixarenes, their unique structure and biological activity make them promising compounds for the development of new anti-cancer therapies. Incorporation of clinically approved active substituted compounds into the basic moiety could enhance the biologically active portion of Calixarenes.

Several Calixarene-based compounds were produced, and their anti-proliferating activity was investigated against various cancer cell lines^4,6^. Inspired by all studies, here, we report a new modified symmetric Calixarene-based compound by loading hydroxyl groups in a para position on both upper and lower rims. The incorporation of the four hydroxyl groups may enhance the hydrogen bonding of the synthesized **HPCR**, and therefore increasing its hydrophilicity. Such a modification in the physicochemical properties may develop an enhanced interaction of **HPCR** with those cell lines which favors hydrophilic interactions. The cytotoxic effect of C-4-Hydroxyphenylcalix[4]resorcinarene (**HPCR**) against breast cancer (MCF7) glioblastoma (U-87 MG) and lung cancer cell lines (A549) has been investigated in this work. Here, we showcase the outcomes of an extensive analysis utilizing in silico technique to evaluate the potential efficacy of **HPCR** as a candidate for inhibiting cancer, suggesting its viability as a therapeutic agent.

Cancer is a leading global health issue, with a significant impact on morbidity and mortality worldwide: Prevalence: As of 2023, approximately 19 million new cancer cases are reported annually, with about 10 million cancer-related deaths each year. The incidence of cancer is expected to increase due to factors such as an aging population and changes in lifestyle. among the Common Types: Breast Cancer: The most common cancer globally, with around 2.3 million new cases annually. Lung Cancer: The leading cause of cancer death, with about 2.2 million new cases each year. Colorectal Cancer: Affects about 1.9 million people annually^7,8^. Prostate Cancer: Approximately 1.4 million new cases each year. Stomach Cancer: Around 1.1 million new cases annually. Despite advancements, existing cancer treatments have limitations and side effects, creating a strong case for exploring new alternatives: 1. Limitations of Current Treatments: Traditional treatments like chemotherapy and radiation therapy can have severe side effects, including damage to healthy cells and long-term health issues. For some cancers, treatments may not be effective or become less effective over time due to drug resistance^2^. Drug Resistance: Many cancers develop resistance to existing therapies, making it challenging to treat advanced stages. Research into new drugs and treatment approaches is crucial to overcoming resistance and improving patient outcomes^3,9^. Personalization Challenges: While precision medicine is advancing, it is not universally available or effective for all cancer types. More research is needed to develop treatments that can be customized to individual patients’ needs and genetic profiles^4^. Access and Affordability: Advanced treatments, such as targeted therapies and immunotherapies, can be prohibitively expensive, limiting access for many patients. Finding cost-effective alternatives could improve access to effective cancer treatments worldwide^5^. Addressing Unmet Needs: Certain cancers, such as pancreatic cancer and glioblastoma, have limited treatment options and poor prognoses. New treatments are urgently needed to address these aggressive cancers and improve survival rates. among the Emerging Alternatives Nanomedicine: Utilizes nanoparticles to deliver drugs directly to cancer cells, minimizing damage to healthy tissues and potentially improving treatment efficacy. Gene Editing: Techniques like CRISPR-Cas^9^ are being investigated to modify cancer cell DNA, offering new ways to target and potentially eradicate tuors. Cancer Vaccines: Both preventive vaccines (e.g., HPV vaccines) and therapeutic vaccines are being developed to stimulate the immune system against cancer cells. Microbiome Research: Investigates how the gut microbiome influences cancer progression and treatment responses, potentially leading to new therapeutic strategies. Artificial Intelligence: AI and machine learning are being used to analyze vast datasets, predict cancer risk, optimize treatment plans, and discover new drug candidates. The need for new alternatives in cancer treatment is driven by the limitations of existing therapies, the challenge of drug resistance, and the demand for more personalized and affordable options^10,11^. Continued research and innovation, such as the development of compounds like C-4-hydroxyphenylcalix[4]resorcinarene, are essential to advancing cancer treatment and improving outcomes for patients worldwide.

In molecular docking studies focused on carcinogenesis, it is crucial to target specific proteins involved in cancer progression. Proteins from cancer cell lines such as U-87 (glioblastoma), MCF-7 (breast cancer), and A549 (lung cancer) have been evaluated due to their roles in signalling pathways that regulate cell proliferation and survival. These proteins, with structures available in the Protein Data Bank (PDB: 3RJ3, 7AXD, 6DUK), are key targets for understanding and disrupting cancer cell growth. Additionally, the human dermal fibroblast cell line (HDF) and its protein structure (PDB: 1CGL) serve as controls to ensure specificity. By elucidating the contribution of these proteins to carcinogenesis, researchers can design targeted therapies that effectively inhibit tumour growth and induce apoptosis in cancer cells.

## Materials and methods

### Chemicals and instrumentation

All of the chemicals and reagents were used without additional purification; they were purchased from Aldrich (Sigma–Aldrich, St. Louis, MO, USA). 1H and 13C NMR spectra were recorded on Gemini Varian-VXR-unity (400, 300 MHz) instrument. Chemical shifts (δ) are reported in ppm downfield from internal TMS standard.

### Synthesis of *C*- 4- Hydroxyphenylcalix[4] resorcinarene (HPCR)

**Fig. 1:**
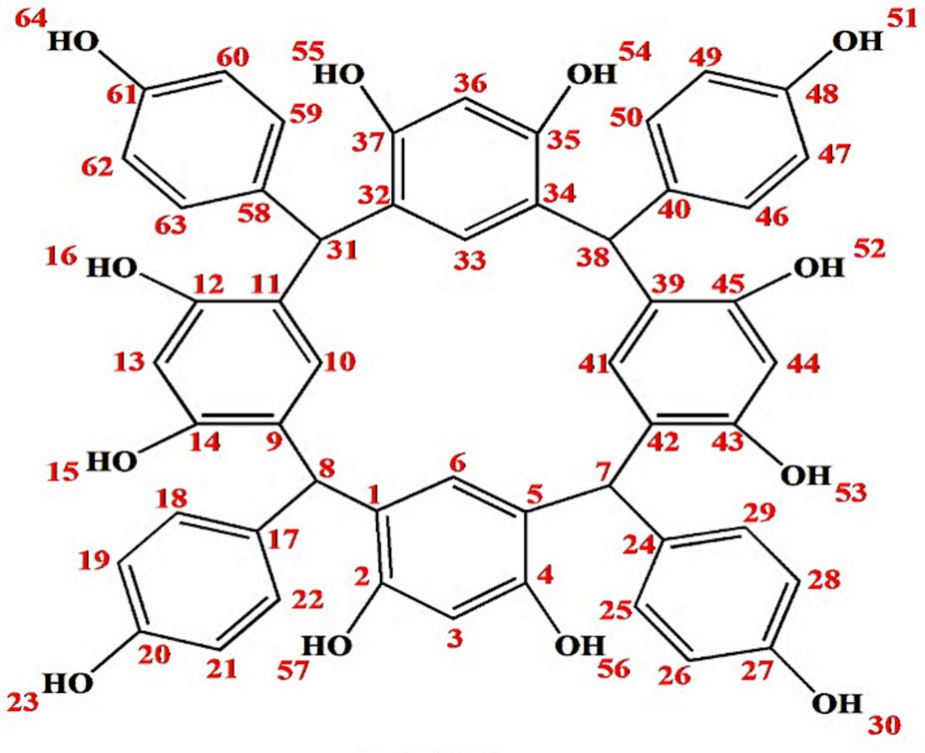
Structure of HPCR.

**Fig. 2:**
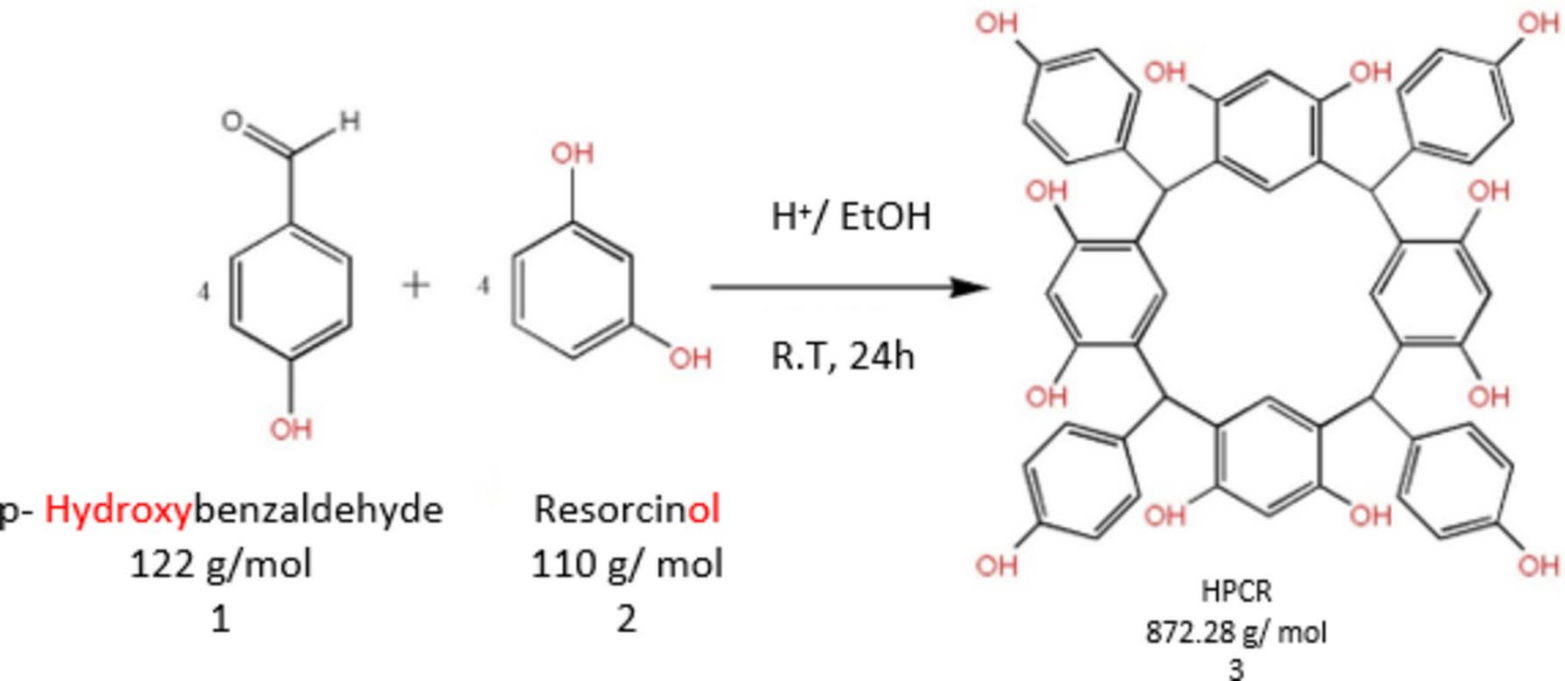
Synthesis of C-4-Hydroxyphenylcalix[4] resorcinarene (HPCR).

^**1**^**H NMR (400 MHz, DMSO-d**_**6**_**)**: δ = 5.43 and 5.52 (s, 4H, H-7, 8, 31, 38), 6.08 and 6.10 (s, 4H. H-3, 13, 36, 44), 6.32 and 6.43 (d, *J*^3^ = 8.0, 8.0 Hz, 8H, H-19, 21, 26, 28, 47, 49, 60,62), 6.49 and 6.50 (s, 4H, H-6, 10, 33, 41), 6.48 and 6.64 (d, *J* ^3^ = 8.0, 8.0 Hz, 8H, H-18, 22, 25, 29, 46, 50, 59, 63), 8.38 and 8.43 (s, 4H, H-23, 30, 51, 64), 8.64 and 8.87 (s, 4H, H-15, 16, 22, 25, 50, 52, 53, 55).

^**13**^**C NMR (100 MHz, DMSO-d**_**6**_**)**: δ = 40.5 (C-7, 8, 31, 38), 101.9 (C-3, 13, 36, 44), 113,9 (C-19, 21, 26, 28, 47, 49, 60, 62), 120.9 (C-1, 5, 9, 11, 32, 34, 39, 42), 129.5 (C-6, 10, 33, 41), 129.7 (C-18, 22, 25, 29, 46, 50, 59, 63), 135.9 (C- 17, 24, 40, 58), 152.5 (C-2, 4, 12, 14, 35, 37, 43, 45), 154.4 (C-20, 27, 48, 61).

The **HPCR** (Fig. 1) was resynthesized as reported previously by Al-Trawneh et al.^12^. The complete procedure and structural elucidation of **HPCR** compound have already been provided in our prior study^13^. HPCR was resynthesized by a single-step organic reaction (Fig. 2**)**. Briefly, an ice-cooled solution of **resorcinol** (2.18 g) and HCl (10 M, 3 mL) was mixed with p-Hydroxybenzaldehyde (2.42 g) dissolved in ethanol (40 ml). The resultant mixture was stirred at room temperature overnight. Afterward, a light pink precipitate was filtered and rinsed several times with 50% aqueous ethanol. The obtained **RESORCINOL** was left overnight in the dark to fully dry. The collected amount was 2.35 g, m.p = 290 °C, (decomp).

**Fig. 3:**
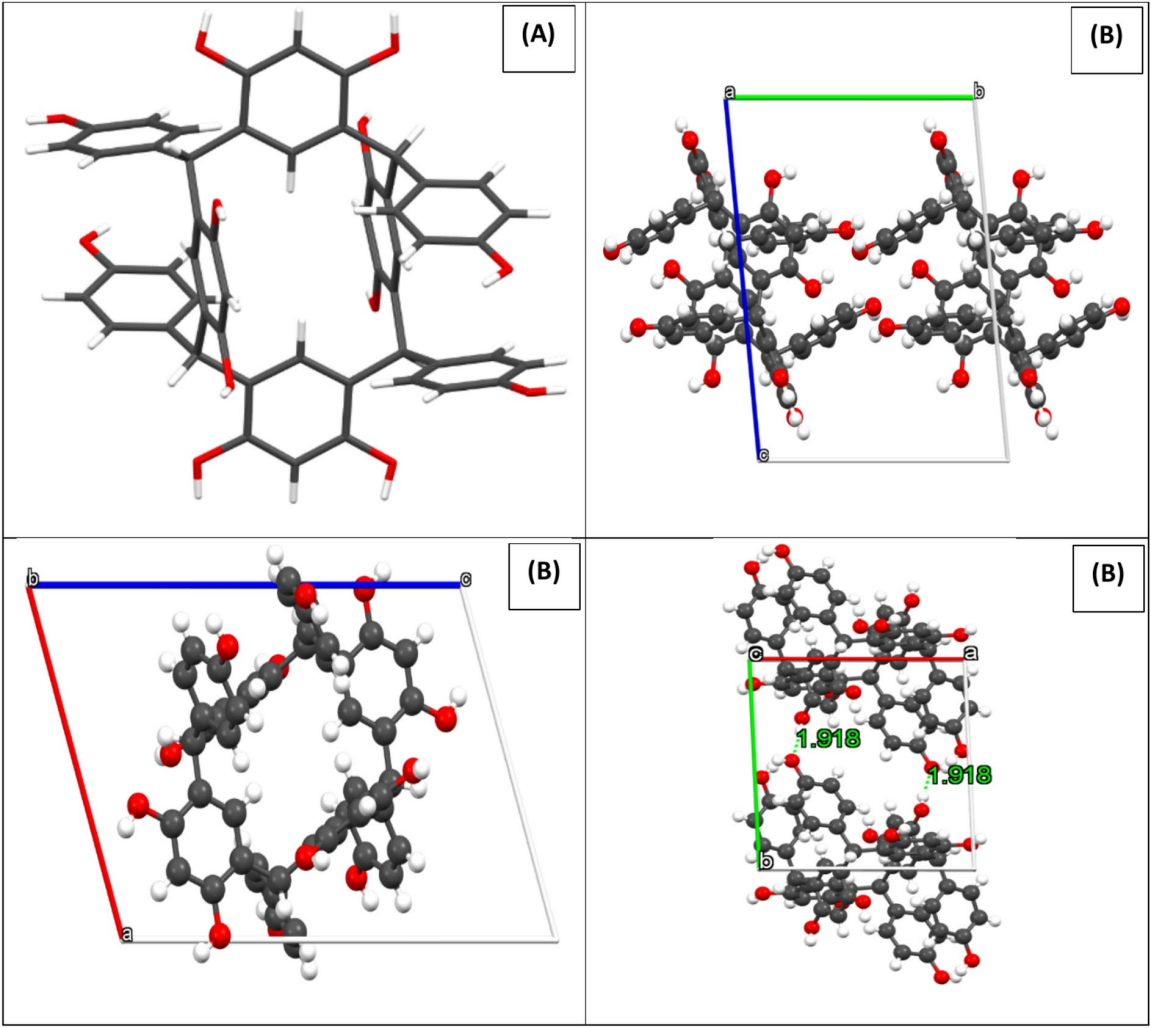
(**A**) The HPCR structure (capped sticks), and (**B**) Projection of the structure along a, b and c axis. Black, red, and snowballs correspond to carbon, oxygen and hydrogen atoms.

X-ray crystallographic analysis indicated that C-4-Hydroxyphenylcalix[4]resorcinarene crystallizes were in the triclinic P-1 (2). It is important to note that the alcohol groups contribute to the production of intermolecular hydrogen bonds in the HPCR compound, which maintains the stability of the crystal network. Figure 3 shows the projections of the structure along the a, b and c axis. with the hydrogen bonds.

### Cell culture

The breast cancer (MCF-7), non-small cell lung cancer (A549), glioblastoma (U-87 MG) and normal human dermal fibroblast (HDF) cell lines were obtained from American Type Culture Collection (ATCC, Manassas, United States). All culture mediums, heat-inactivated fetal Calf serum (FCS), L-glutamine, sodium pyruvate and penicillin- streptomycin antibiotics (P-S) were obtained from Capricorn Scientific GmbH, Ebsdorfergrund, Germany. The MCF-7 and A549 cell lines were cultured and maintained in a Roswell-Park Memorial Institute medium (RPMI 1640) containing 2 mM of L-glutamine, 10% (v/v) of FCS, 1 mM of sodium pyruvate and 1% of (P-S). Moreover, the (U-87 MG) and (HDF) were cultured and maintained in Dulbecco’s Modified Eagle Medium (DMEM) containing 2 mM of L-glutamine, 10% (v/v) of FCS, 1 mM of sodium pyruvate and 1% of (P-S). All these cell lines were kept in the tissue culture incubator at 37 ºC in a 5% CO_2_ humidified atmosphere.

### Cytotoxicity assay

Thiazolyl Blue Tetrazolium Bromide (MTT) assay was performed to determine the anti-proliferative activity of the HPCR, as mentioned previously^14–16^. After harvesting of cells, cells were inoculated in a round bottom 96-well plate (Corning, USA) at a density of 1 × 10^4^ cells/ml and incubated overnight at 37 °C in a 5% CO_2_ atmosphere, to allow cell adherence. Cells were then incubated with HPCR compound (**1, 50, 100, 200, 400, 600, 800,** and **1000** μM/ mL). The anti-cancer drug cisplatin (Sigma-Aldrich Chemie GmbH, Germany) was used as a positive control with different concentrations of 1, 10, 50, and 100 μM/mL in all experiments. After 96 h of cell treatment, the medium was aspirated, and 0.2 mL MTT at a concentration of 5 mg/mL (Sigma-Aldrich Chemie GmbH, Germany) was added to each well for 4 h. Finally, the supernatant was removed and the formed formazan crystals were dissolved in 0.15 mL Dimethyl sulfoxide (DMSO, Sigma-Aldrich Chemie GmbH, Germany). After that, the absorbance of formazan solutions was measured at 550 nm using BioTek Synergy HTX multimode readers (Agilent Technologies, CA, USA).

The Half-maximum inhibitory concentration (IC_50_) to HPCR was used to determine the 50% cell growth inhibitory effect of HPCR against various normal and cancer cell lines. The IC_50_ was calculated by non-linear regression method for each cell line via GraphPad Prism 7 Software, California, USA. Additionally, the degree of HPCR to induce selective cytotoxicity toward cancer cells was determined by the selectivity index (SI). It is the ratio of the inhibitory effect of HPCR on human normal cells (HDF) to the inhibitory effect on cancer cells (A549, U-87 MG, and MCF-7). SI more than 3, indicates that HPCR has selectivity towards particular cancer cells than normal cells^17,18^.

### Morphological analysis

Morphological analysis was performed with some modifications as previously stated^19–21^. Cells were inoculated at the density of 1 × 10^4^ cells/ml (180 μL/well) in a flat bottom 96-well plate (Corning, USA). After overnight incubation to allow cell adherence, cells were treated with HPCR and cisplatin at the same concentrations as described earlier. Cells were also treated with HPCR and cisplatin at the respective IC_50_ for each cell line. Afterward, the treated cells were stained with hematoxylin after fixation with ice-cold methanol (4 °C) for 30 min. Plates were viewed under a Zeiss Axiovert 40C inverted microscope and digital images were captured using ToupCam digital camera.

### In silico predication of toxicity

The online ProTox-II webserver was utilized to assess the toxicity of the synthesized HPCR (https://tox.charite.de/protox3/index.php?site=compound_input, accessed on 20 August 2024). This tool predicts acute oral toxicity in rodents as function of median lethal dose (LD50) and classifies compounds into six toxicity classes ranging from class I (very toxic) to class VI (non-toxic). It further incorporates models for prediction of organ toxicity, different toxicity endpoints, toxicological targets and pathways, compound metabolizing enzymes and finally molecular initialling events behind toxic effect^22,23^.

### Statistical analysis

The statistical packages for social sciences (SPSS) program version 19 was used to analyze all variables. For each experimental run, the data were presented as average ± standard deviation (SD). The statistical significance of the differences in the means of the three experiments was assessed using the *t-test*. If the p-value was less than 0.05, a statistical difference was regarded as significant.

### Technique of molecular operating environmental‑docking (MOE)

The MOE docking approach module (Vs. 2015) represents the latest software in the realm of docking simulation techniques, specifically designed to assess the inhibitory potential against cancerous proteins^24^. The selected proteins for evaluation included cancer cell lines (U-87, MCF-7, A549) and the human dermal fibroblast cell line (HDF). The respective protein structures were obtained from the Protein Data Bank (PDB: 3RJ3), (PDB: 7AXD), (PDB: 6DUK), and (PDB: 1CGL). The simulation technique was applied to C- 4- Hydroxyphenylcalix [4] resorcinarene (HPCR) and the standard drug Cisplatin obtained from the PubChem database (http://pubchem.ncbi.nlm.nih.gov), commencing with orientation and progressing to optimization for the tested compound^25^. Pre-optimization involved configuring the compounds by adding hydrogen atoms, atomic charges, and potential energies, with adjustments made using the MMFF94x force field during energy minimization^26^. The orientation of proteins was a key focus, beginning with the placement of hydrogen atoms over receptors after removing water molecules. The subsequent stages included connecting receptor types, fixing potential energies, and conducting site-finder analysis over the line helix of protein amino acids. Dummies were adjusted over alpha-sites in the protein, paving the way for the initiation of the docking process. The docking simulation process varied in duration for all tested compounds, but each process comprised an average of 30 poses. Various features, such as ligand type, receptors, interaction type, H-bond length, and energy content, were recorded for the docked complexes^27,28^. Furthermore, interaction patterns and surface maps were extracted to validate comparative features.

### Density functional theory approach

Several theoretical approaches have been used to study the local and global reactivity of various organic and inorganic compounds in different fields such as synthesis, medicine corrosion…..etc.^29^. To examine these features, density functional theory (DFT), has been essential. Furthermore, to optimize the structure of the anticancer drug HPCR, we applied the basic set of digital double polarization plus (DNP) and GGA/PBE functions in Materials Studio software using DMol^3^ methodologies. HOMO and LUMO energies, energy gap (ΔEgap), electron affinity (EA), ionization potential (IP), electronegativity (χ), percentage of electrons transported (ΔN), and dipole moment characteristics (μ), are among the theoretical parameters that were calculated using Eqs. 1 to 7 refs.^30,31^. In addition, these theoretical tools facilitated the identification of several isosurfaces using Multiwfn, VMD, and Gnuplot, including the optimal structure, HOMO and LUMO isosurfaces, as well as ELF, LOL, NCI, and RDG isosurfaces. We used Hirshfeld population analysis to calculate Fukui indices (Fukui ( +) and Fukui (−)). To predict electrophilic and nucleophilic sites. These parameters were calculated using Eqs. 8 and 9 ref.^32^. Additionally, to investigate the interactions between the anticancer drug HPCR and four proteins (PDB: 3RJ3, PDB: 7AXD, PDB: 6DUK, and PDB: 1CGL), we optimized their structures using the Forcite technique in Materials Studio software^33^.

1 $$\Delta E_{gap} = E_{LUMO} - E_{HOMO} = \,{\text{IP}} - {\text{EA}}$$

2 $$IP={-E}_{HOMO}$$

3 $$EA=-{E}_{LUMO}$$

4 $${\chi }_{inh}=\frac{IP+EA}{2}$$

5 $${\eta }_{inh}=\frac{{\Delta E}_{gap}}{2}$$

6 $$\mu =\frac{{E}_{LUMO}+{E}_{HOMO}}{2}$$

7 $${\Delta N}_{max}=\frac{-\mu }{{\eta }_{inh}}$$

8 $${\text{f}}_{{\text{k}}}^{ + } = {\text{q}}_{{\text{k}}} \left( {{\text{N }} + { 1}} \right) - {\text{q}}_{{\text{k}}} \left( {\text{N}} \right)$$

9 $${\text{f}}_{{\text{k}}}^{ - } = {\text{q}}_{{\text{k}}} \left( {\text{N}} \right) - {\text{q}}_{{\text{k}}} \left( {{\text{N}} - {1}} \right)$$

The electron concentrations on atom k that correspond to N+1, and N−1 are represented by the symbols qk (N+1), qk (N), and qk (N−1). Systems with N+1, N, and N−1 electrons, respectively.

## Results and discussion

### NMR analysis

#### Structural elucidation of HPCR compound

The structure of HPCR is supported by NMR spectral data, given in the experimental section, are in accordance with the suggested structures. Assignments of the 1H- NMR and ^13^C- NMR signals to different protons and carbons are based on DEPT-90 and DEPT-135.

**Fig. 4:**
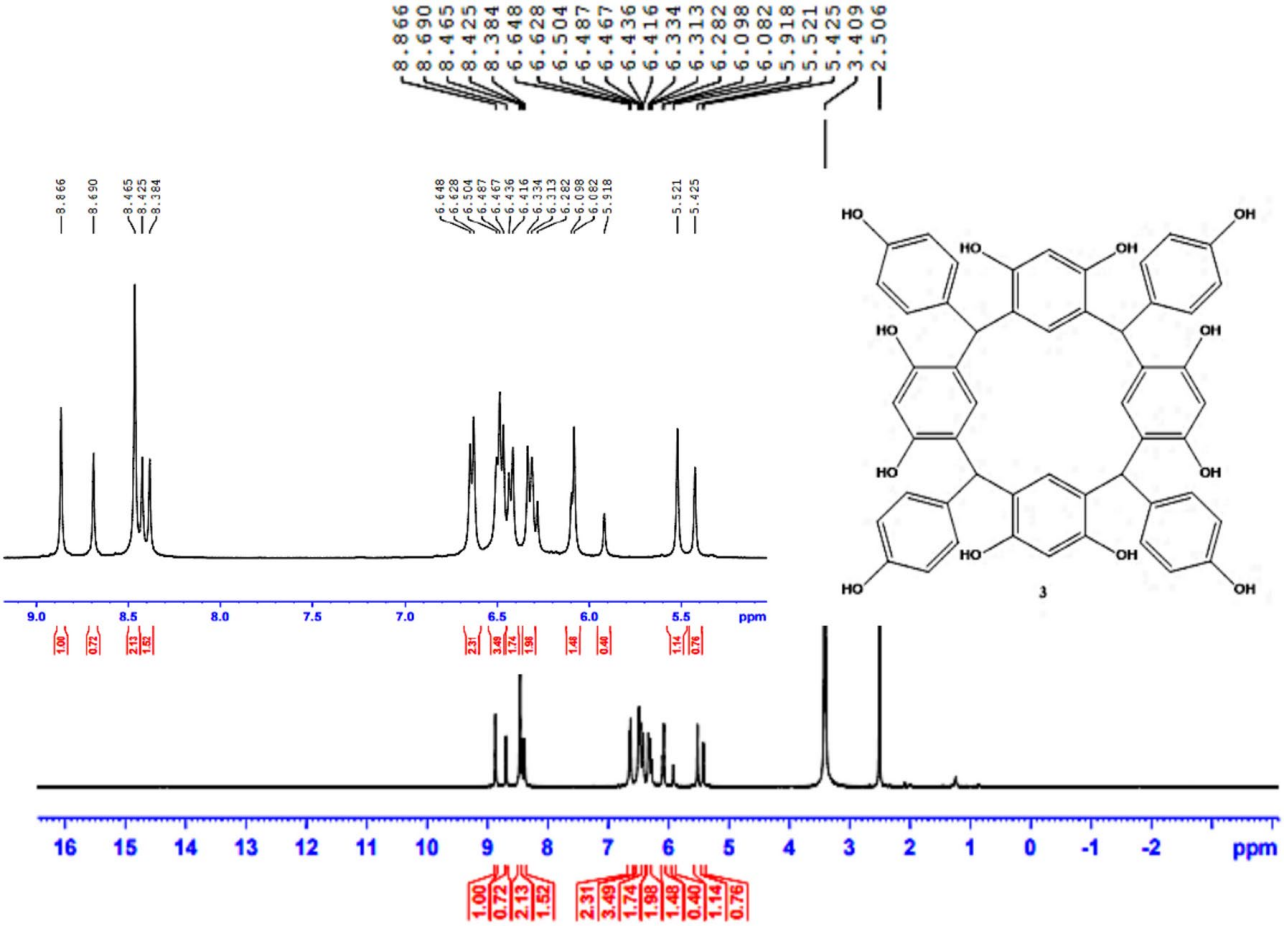
^**1**^H-NMR spectrum of HPCR (400 MHz, DMSO-d_**6**_).

The ^**1**^H-NMR spectrum showed two signals for each type of atom, and this is attributed to the presence of two conformers (boat and chair conformers). Thus, the spectrum showed two singlet signals at 5.43 and 5.52 ppm, which are assigned to the H-7, 8, 31, and 38 protons. Other singlet signals appeared in the aromatic region at 6.08 and 6.10 ppm which is attributed to H-3, 13, 36, 44 protons. Additionally, two singlet signals in the aromatic region appeared at 6.49 and 6.50 ppm, which is assigned to H-6, 10, 33, and 41 protons. The H-23, 30, 51, and 64 protons in HPCR moiety resonate with two singlet signals which resonate at 8.38 and 8.43 ppm. Similarly, H-15, 16, 22, 25, 50, 52, 53, and 55 display significant downfield shifts at 8.64 and 8.87. Moreover, the ^1^H-NMR spectrum showed a doublet signal at 6.32 and 6.43 ppm, which are assigned to H-19, 21, 26, 28, 47, 49, 60, 62 with a *J*^3^ = 8.0, 8.4 Hz. Other doublet signals at 6.48 and 6.64 ppm are assigned to H-18, 22, 25, 29, 46, 50, 59, 63 with a *J*^**3**^ = 8.0, 8.0 Hz as shown in (Fig. 4).

**Fig. 5:**
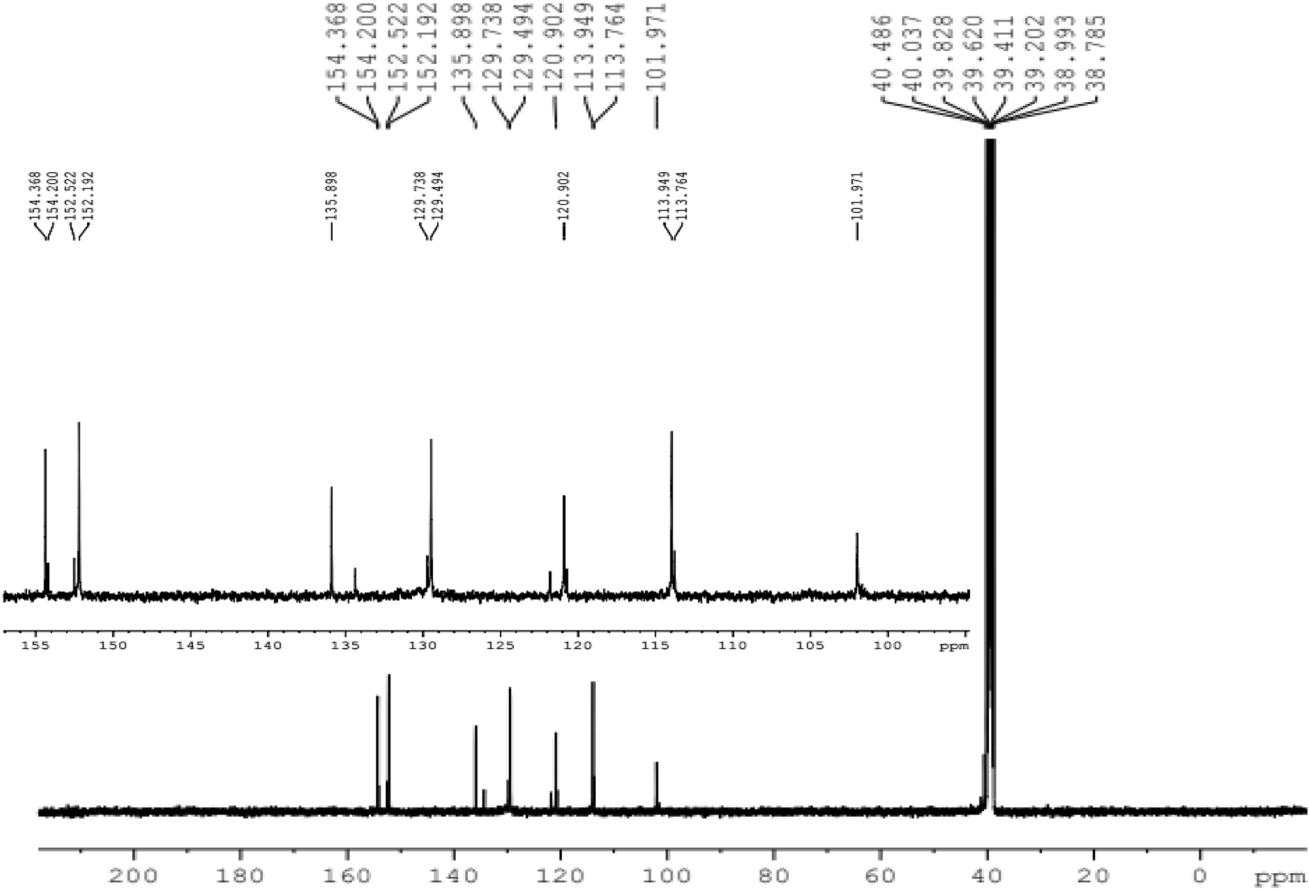
^**13**^C-NMR spectrum of HPCR (100 MHz, DMSO-d_**6**_).

The ^**13**^C-NMR spectrum of HPCR revealed clearly that all various carbon atoms are detectable as shown in Fig. 5. The peak of H-(C)7, 8, 31, 38 resonates in the alkane’s region at 40.5 ppm. Afterward, all the peaks appeared in the alkenes and aromatic compounds region, as the C** = **(C)3, 13, 36, 44 appears at 101.9 ppm, and C** = **(C)19, 21, 26, 28, 47, 49, 60, 62 shown at 113.9 ppm. Also, there is a peak that appeared at 120.9 which is attributed to alkene groups C** = **(C)1, 5, 9, 11, 32, 34, 39, 42. The peaks of C** = **(C)6, 10, 33, 41 and C** = **(C)18, 22, 25, 29, 46, 50, 59, 63 resonated at 129.5 and 129.7, respectively. There is a peak that showed at 135.9 ppm which is assigned to HC-(C)17, 24, 40, 58. Furthermore, two broad peaks appeared at 152.5 and 154.4 ppm which is attributed to HO-(C)2, 4, 12, 14, 35, 37, 43, 45 and HO-(C)20, 27, 48, 61, respectively, and the broadness indicates to the symmetry of OH- groups.

**Fig. 6:**
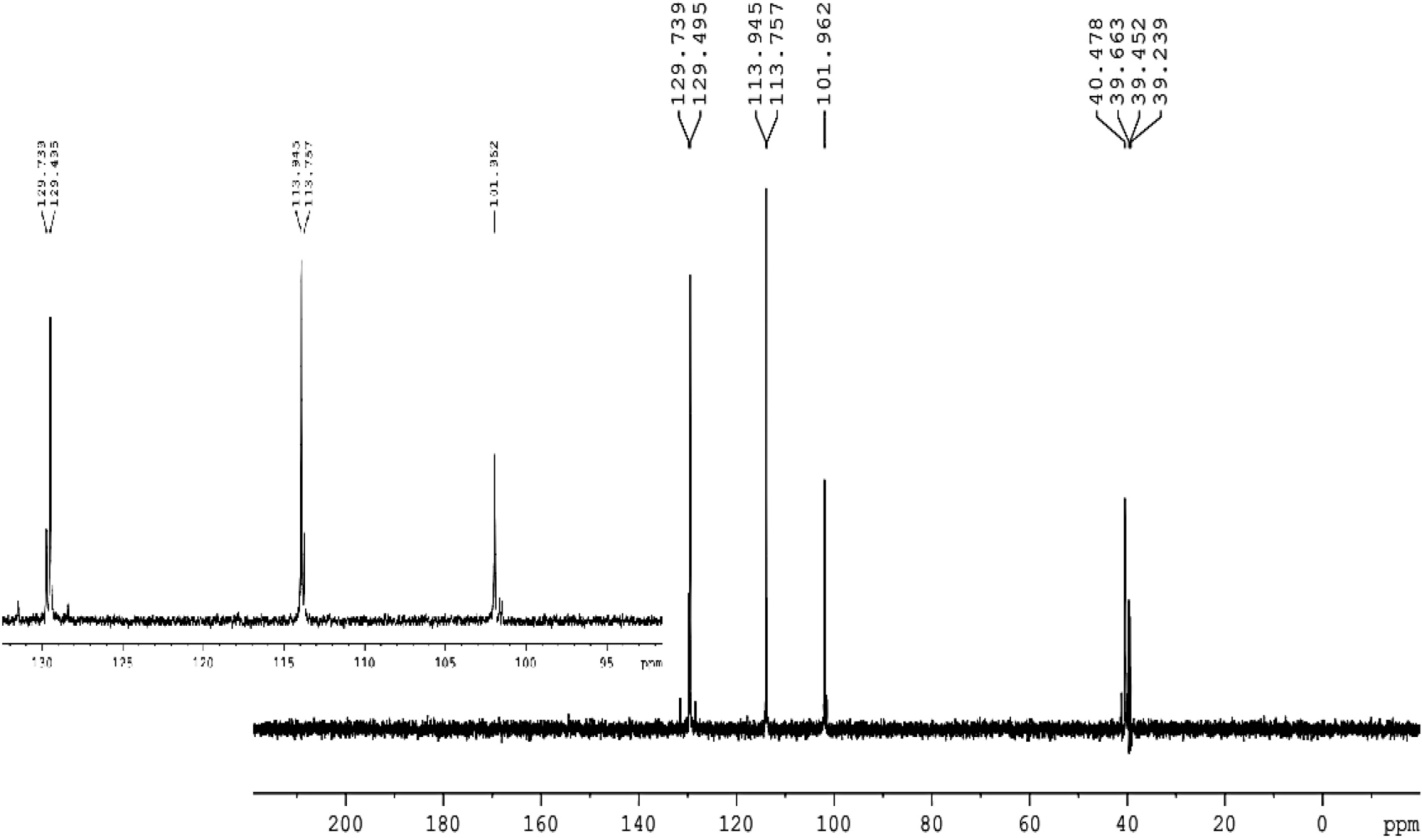
DEPT-90 spectrum of HPCR (DMSO-d_**6**_).

**Fig. 7:**
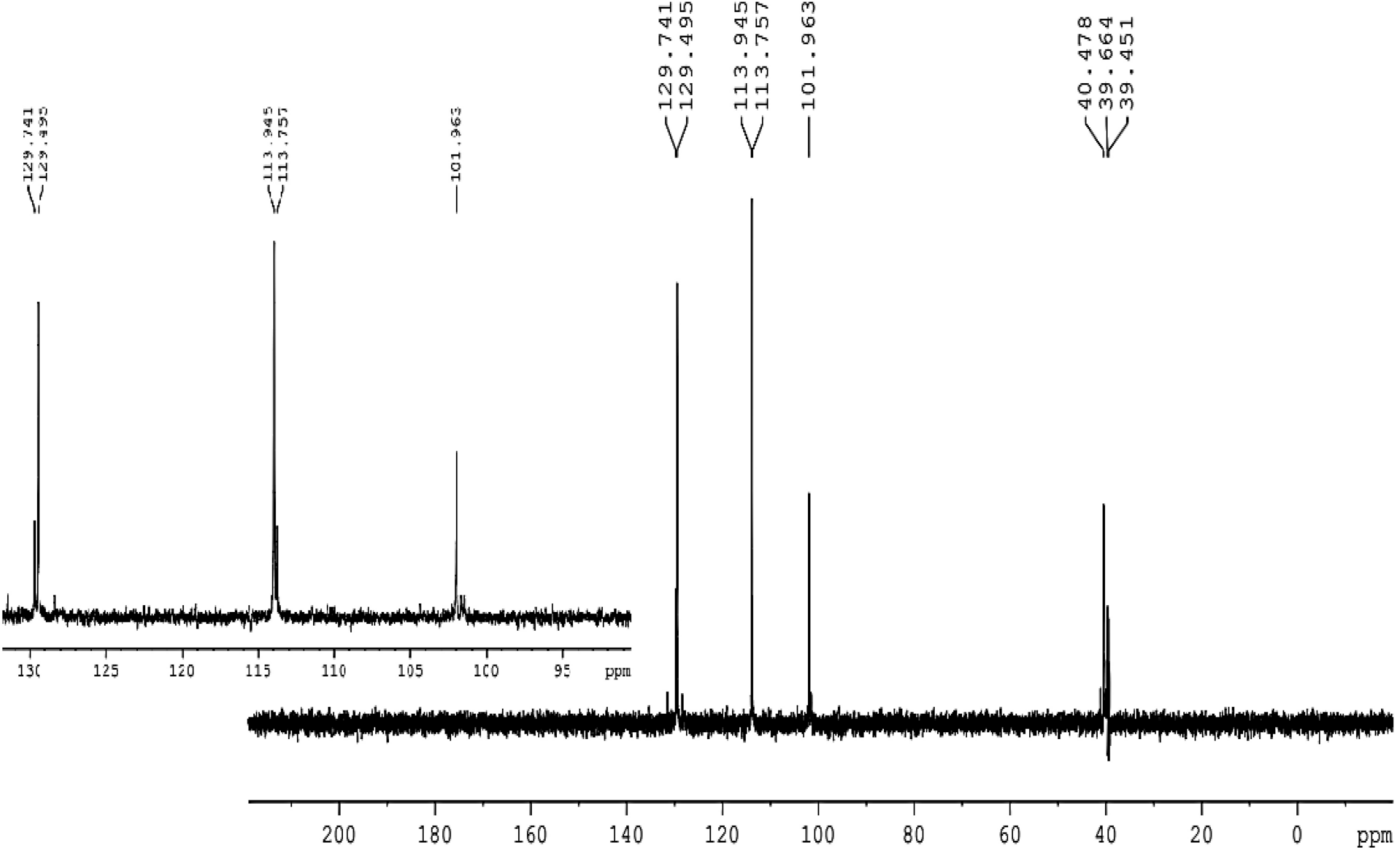
DEPT-135 spectrum of HPCR (DMSO-d_**6**_).

From the DEPT-90 and DEPT-135 spectrum, several signs of CH carbons appear, as shown in (Fig. 4). Conversely, no negative signals appeared in the DEPT-135 spectrum, confirming the absence of CH2 groups. Therefore, consistent with the suggested structure of HPCR. ^**1**^H- NMR, ^**13**^C- NMR, DEPT-90 and DEPT-135 spectra for HPCR are shown in (Figs. 4–7).

### Cytotoxicity assay

**Fig. 8:**
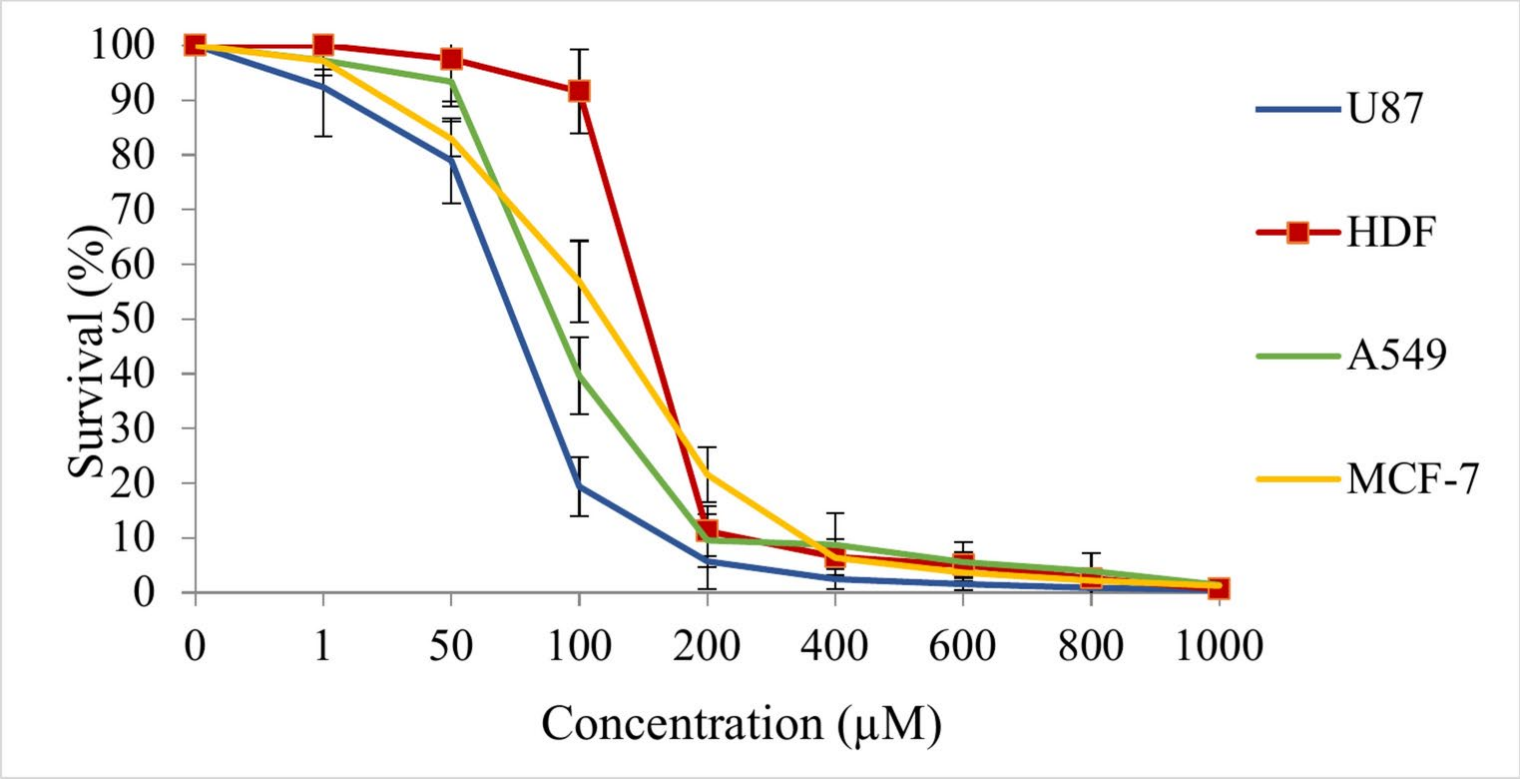
Anti-proliferative activity of HPCR on normal cells; human dermal fibroblast (HDF) and cancerous cell lines including breast cancer (MCF-7), non-small cell lung cancer (A549) and glioblastoma (U-87 MG). Cells were treated with HPCR at various concentrations ranging from 1–1000 μM for 96 h.

Herein, HPCR exhibited a significant concentration-dependent growth inhibitory effect on normal and cancer cell lines (p ≤ 0.05), as shown in Fig. 8, Table 1. The most growth inhibitory effect was displayed against U-87 MG and A549 cell lines [IC_50_ = 71 ± 10 μM.ml^-1^ (U-87 MG), and 92.3 ± 7.3 μM.ml^-1^ (A549)] compared to the HDF cell line [ IC_50_ = 146.7 ± 12 μM.ml^-1^ (HDF), (p ≤ 0.05)]. Compared to standard drug cisplatin, HPCR showed moderate growth inhibitory activity against U-87 MG and A549 cell lines. On the other hand, HPCR showed very low activity towards the MCF-7 cell line [IC_50_ = 112.3 ± 5.4 μM.ml^−1^, (p ≤ 0.05)] compared to that reported for cisplatin towards the same cell line [IC_50_ = 26.9 ± 4.7 μM.ml^−1^].

**Table 1:** The median inhibitory concentration** (**IC_50,_ μM) and selectivity index (SI) (mean ± SD) values of cancer cell lines including breast cancer (MCF-7), non-small cell lung cancer (A549) and glioblastoma (U-87 MG) as well as normal cells; human dermal fibroblast (HDF) for HPCR and Cisplatin upon 96 h treatment period.

	IC_50_ (μM)				SI		
Drug/cell line	HDF	U-87	A549	MCF-7	U-87	A549	MCF-7
HPCR	146.7 ± 12	71 ± 10	92.3 ± 7.3	112.3 ± 5.4	2.07	1.58	1.31
Cisplatin	40.9 ± 5.4	28.3 ± 5.3	30.4 ± 5.2	26.9 ± 4.7	1.44	1.34	1.5

Based on the data shown in Table 1, these results were reflected on selectivity where there was no any selectivity seen towards any cancer cell line. Interestingly, a significant selective growth inhibitory activity towards U-87 MG and A549 cell lines was found at a concentration of 100 μM compared to the normal cell line (p ≤ 0.05). One explanation for our results may be attributed to the full symmetry of the HPCR and the presence of the hydroxyl groups in the para position on both the upper and lower rims. It has been shown that different substitutions at phenylcalix [4] resorcinarene chemical ore largely reduced the cytotoxic potency of resulting compounds against normal and cancer cell lines^21,34,35^. For instance, the nitro-substituted C- phenylcalix [4] resorcinarene derivatives showed no cytotoxicity against human cell lines with approximate IC_50_ values of 5 mg/mL^21^. Overall, despite the low selectivity seen at most concentrations, it is worth investigating HPCR activity in a wide spectrum of cell lines at 100 μM concentration.

### Morphological analysis

**Fig. 9:**
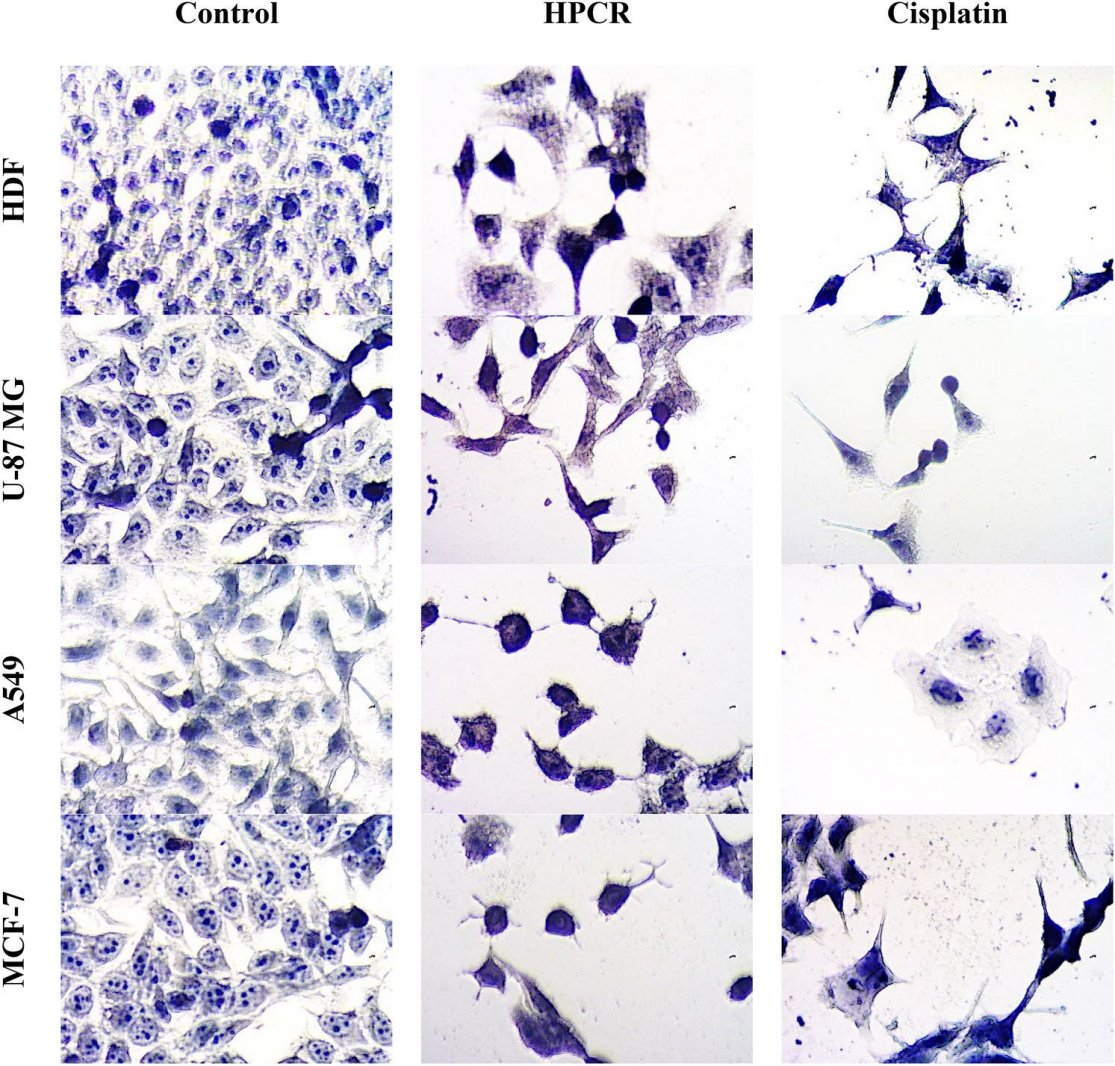
Cytotoxic effects of HPCR and Cisplatin against normal cells; human dermal fibroblast (HDF) and cancerous cell lines including breast cancer (MCF-7), non-small cell lung cancer (A549) and glioblastoma (U-87 MG) upon 96 h treatment period. The treatment was carried out at the respective IC_50_ of HPCR for each cell line (HDF; 146.7 ± 12, MCF-7; 112.3 ± 5.4, A549; 92.3 ± 7.3 and U- 87 MG; 71 ± 10) as well as cisplatin (HDF; 40.9 ± 5.4, MCF-7; 26.9 ± 4.7, A549; 30.4 ± 5.2and U- 87 MG; 28.3 ± 5.3) (40 × objective lens, total magnification = 400x).

Many morphological alterations were determined in cells treated by HPCR and the standard drug cisplatin, as illustrated in Fig. 9 These changes indicate a disturbance of the cell cytoskeleton. Untreated cancer cells had an atypical spindle shape, where the nucleus was uniformly and heavily stained than cytoplasm. Treatment with HPCR separated cells from each other and cells became rounded, shrinkaged and underwent nuclear condensation. These manifestations are similar to those caused by cisplatin treatment implying cell apoptosis pathway^36,37^.

### In silico predication of toxicity

Based on ProTox-II predictions, the toxicity profile showed that HPCR was assigned to toxicity class V for acute oral toxicity (may be harmful if swallowed, 2000 < LD_50_ ≤ 5000), with an estimated LD50 of 3430 mg/kg. This indicates that the HPCR is non-toxic as the predicated toxicity class covers a very high concentration range, compared to the identified IC_50_ values. Moreover, the HPCR showed a favourable toxicity profile with no hepatotoxicity, neurotoxicity and cardiotoxicity. Additionally, the HPCR demonstrated no potential for induction of carcinogenicity, mutagenicity, cytotoxicity, ccotoxicity, clinical toxicity, and nutritional toxicity. However, other toxicities like immunotoxicity, nephrotoxicity and respiratory toxicity were considered less probable events as the confidence limit values for these toxicities were less than 0.7 and need more research to provide confirmation. Such less probable toxicities might be initiated through signalling pathways of Aryl hydrocarbon Receptor (AhR), Estrogen Receptor Alpha (ER), Mitochondrial Membrane Potential (MMP), induction of Transtyretrin (TTR) event and drug metabolizing enzymes of Cytochrome CYP2C19 and CYP2C9. Therefore, HPCR has a favourable safety profile and stands out as promising starting compound for development of safer therapeutic options.

### Molecular docking analysis

**Fig. 10:**
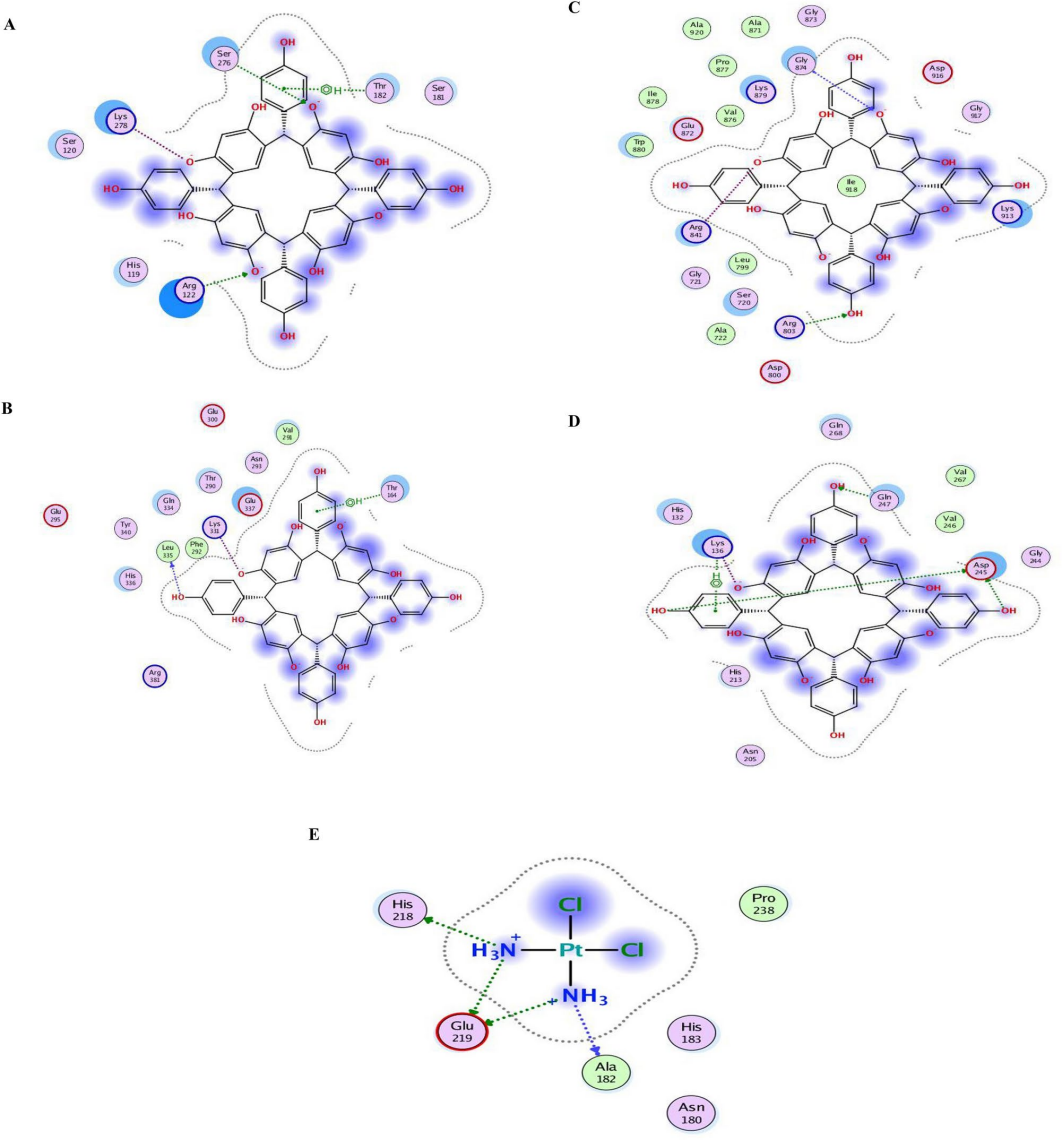
Best interaction patterns for HPCR tested complexes against 3RJ3 (**A**), 7AXD (**B**), 6DUK (**C**), 1CGL (**D**) and cisplatin (inhibitor) with 1CGL.

A recognized and contemporary bioinformatics technique that serves as a valuable tool in medicine and biology is Molecular Docking. This method facilitates the simultaneous comparison and analysis of thousands of substances using Virtual Screening Libraries. The assessment is based on the comparison of binding energy scores associated with a specific target protein^38^. The docking results for HPCR and the standard drug Cisplatin with various cancer cell lines (U-87, MCF-7, A549) and the human dermal fibroblasts cell line (HDF) are presented, indicating the estimated binding energy scores (Kcal mol^-1^). A more negative score indicates a stronger binding affinity between the ligands and targets. Table 2 highlights the optimal docking results, including different bond interactions within the ligand-protein complexes. The 2D and 3D ligand–protein images clearly demonstrate the effective penetration of all compounds into the active binding site within the protein’s cavity, as illustrated in (Fig. 10).

**Table 2:** Docking interaction parameters for C-4-Hydroxyphenylcalix[4] resorcinarene (HPCR) against four selected proteins.

Proteins	S (energy score)	RMSD_Refine*	Interacting amino acid	Type of interaction bond
3RJ3	-7.1127	1.0597	SER276 THR182 ARG122	H-acceptor pi-H H-acceptor
7AXD	-6.1856	0.9183	LEU335 THR182 LYS331	H-donor pi-H Ionic
6DUK	-7.3611	1.224	ARG803 ARG841 GLY874	H-acceptor Ionic H-acceptor
1CGL	-5.6105	1.619	GLU247 ASP245 LYS136 LYS136	H-donor H-donor Ionic pi-H

*RMSD_Refine: the root-mean-squared-deviation (RMSD) between the predicted pose and those of the crystal one (after and before refinement process, respectively).

The simulations revealed that HPCR demonstrated higher binding energy values compared to the standard drug Cisplatin in the Site pocket of the proteins (PDB: 3RJ3), (PDB: 7AXD), (PDB: 6DUK), and (PDB: 1CGL). Specifically, HPCR exhibited high binding energy values of −7.1127 kcal/mol, −6.1856 kcal/mol, −7.3611 kcal/mol, and −5.6105 kcal/mol, respectively. However, the binding scores of cisplatin with the four proteins did not exceed -4.8747 kcal/mol, which was observed with the protein from the human dermal fibroblast cell line (HDF, PDB: 1CGL). Cisplatin exhibited interactions through one hydrogen bond acceptor interaction with ALA182 and three hydrogen bond donor interactions with the amino acid residues GLU219 and HIS201. These interactions demonstrate cisplatin’s relatively moderate binding affinity with these proteins, highlighting the specificity of its interactions with other targets. The findings from our docking simulations hold promise for informing and guiding future in vitro experiments and clinical assessments targeted at cancer cell lines, including U-87, MCF-7, and A549, as well as the human dermal fibroblasts cell line (HDF). These results provide a foundation for further exploration and validation in laboratory settings, potentially offering valuable insights into the effectiveness of compounds like C-4-Hydroxyphenylcalix[4]resorcinarene (HPCR) in combating cancer. The differential binding energies observed in our simulations, particularly the higher binding energy values of HPCR compared to the standard drug Cisplatin, underscore the potential significance of HPCR in targeting specific protein sites associated with cancer. Subsequent in vitro experiments and clinical evaluations can build upon these docking results to explore the practical applications and therapeutic potential of HPCR in cancer treatment.

### Global and local reactivity results

**Fig. 11:**
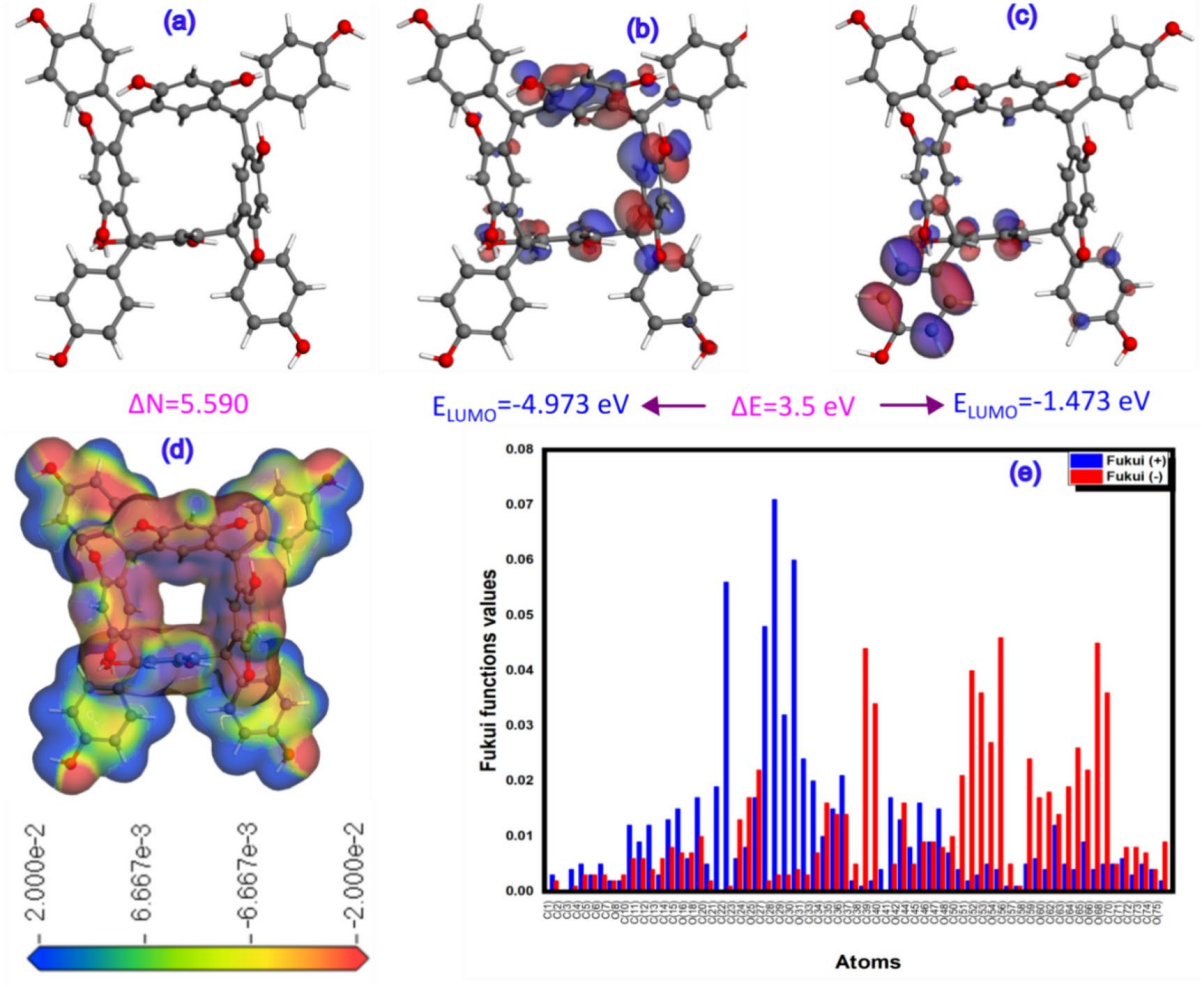
Optimized structure (**a**), HOMO (**b**), LUMO (**c**), ESP_map_ (**d**), Fukui functions indices of the HPCR molecule derived by using the DFT/GGA tool.

**Fig. 12:**
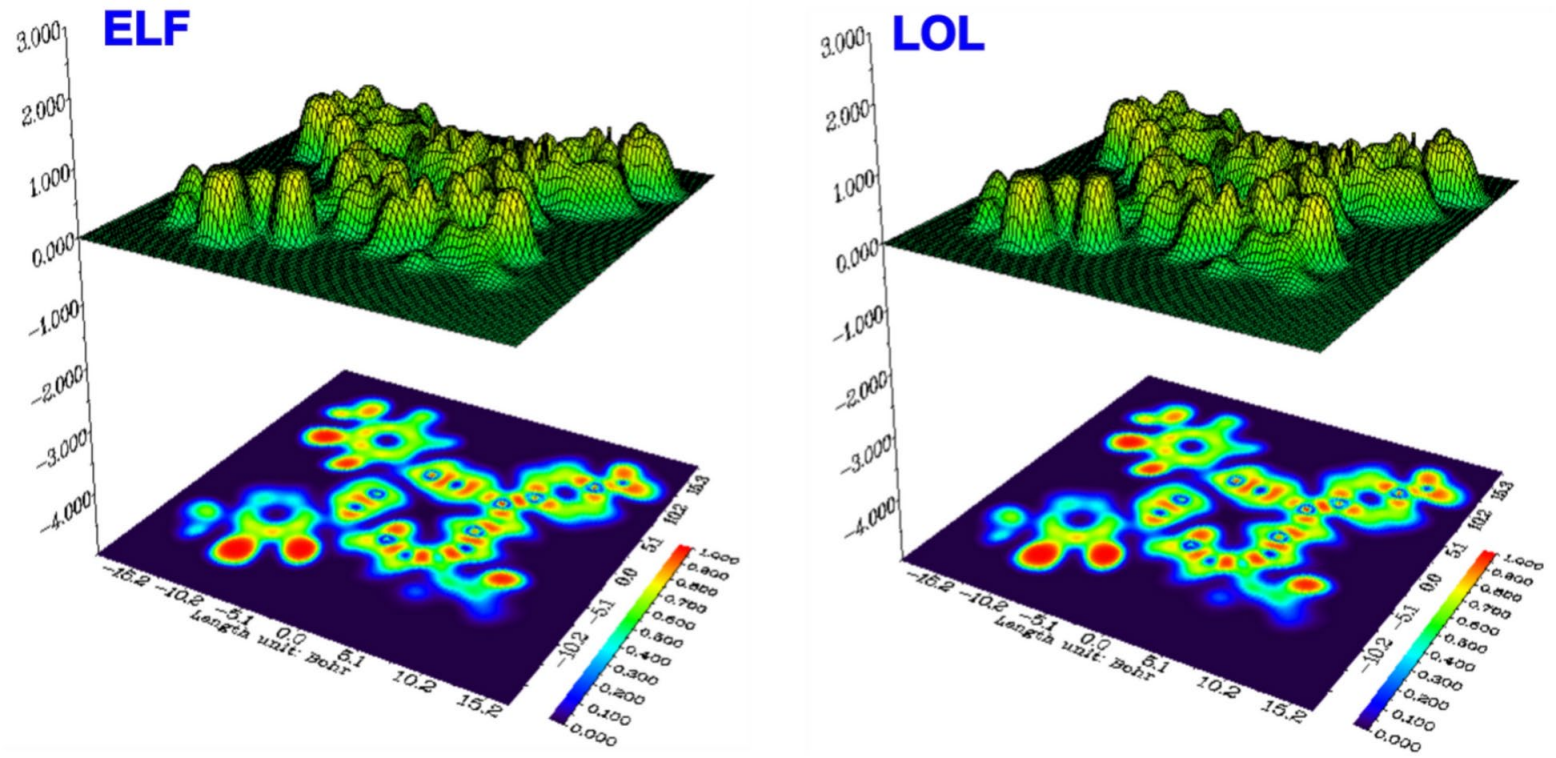
Electron localization functions (ELF), and localized orbital locator (LOL) of the HPCR molecule found by using DFT/GGA technology.

Density functional theory (DFT) is an indispensable tool for estimating global and local reactivity through the use of various quantum parameters and the extraction of important isosurfaces such as the most occupied molecular orbitals (HOMO), the least occupied molecular orbitals (LUMO), the electronic localization function (ELF) and the localized orbital locator (LOL). Fukui indices can also be calculated^39^. Figure 11 illustrates these results in this context, with Fig. 11a the optimal geometry, Fig. 11b the HOMO, Fig. 11c the LUMO, Fig. 11d the ESP map, and Fig. 11e the Fukui function indices of the HPCR molecule. The HOMO and LUMO isosurfaces show that the electron distribution in HPCR molecules is centered on the benzene ring, which is the active nucleus of the electron cloud in this molecule^40^. Furthermore, the ESP isosurface of the HPCR structure displays both negative and positive charges, indicating the presence of electrophilic and nucleophilic sites capable of accepting and donating electrons to various proteins such as (PDB: 3RJ3), (PDB: 7AXD), (PDB: 6DUK) and (PDB: 1CGL)^41^. The fact that the electron transfer number (ΔN_max_) in Fig. 11 is positive indicates that the molecule is electrophilic, i.e. it prefers to absorb electrons from other species and receive them rather than lose them, confirming this hypothesis. Figure 12 shows the existence of numerous atoms, including oxygen at positions 54, 66, and 68, and carbon at positions 39, 40, 50, and 52. These atoms constitute the nucleophilic sites of the HPCR molecule^32^. On the other hand, the electrophilic sites of the HPCR molecule are also occupied by the carbon atoms at positions 22, 23, 27, 28, and 30^32^. The Electron Localization Function (ELF) and the Localized Orbital Locator (LOL) are used to validate the existence of many positions and to validate previous results. Figure 12 illustrates the iso surfaces of these results, ELF and LOL. In particular, the presence of red and blue colors confirms the electrophilic and nucleophilic positions^42^.

### Adsorption of HPCR compound 1CGLprotein

**Fig. 13:**
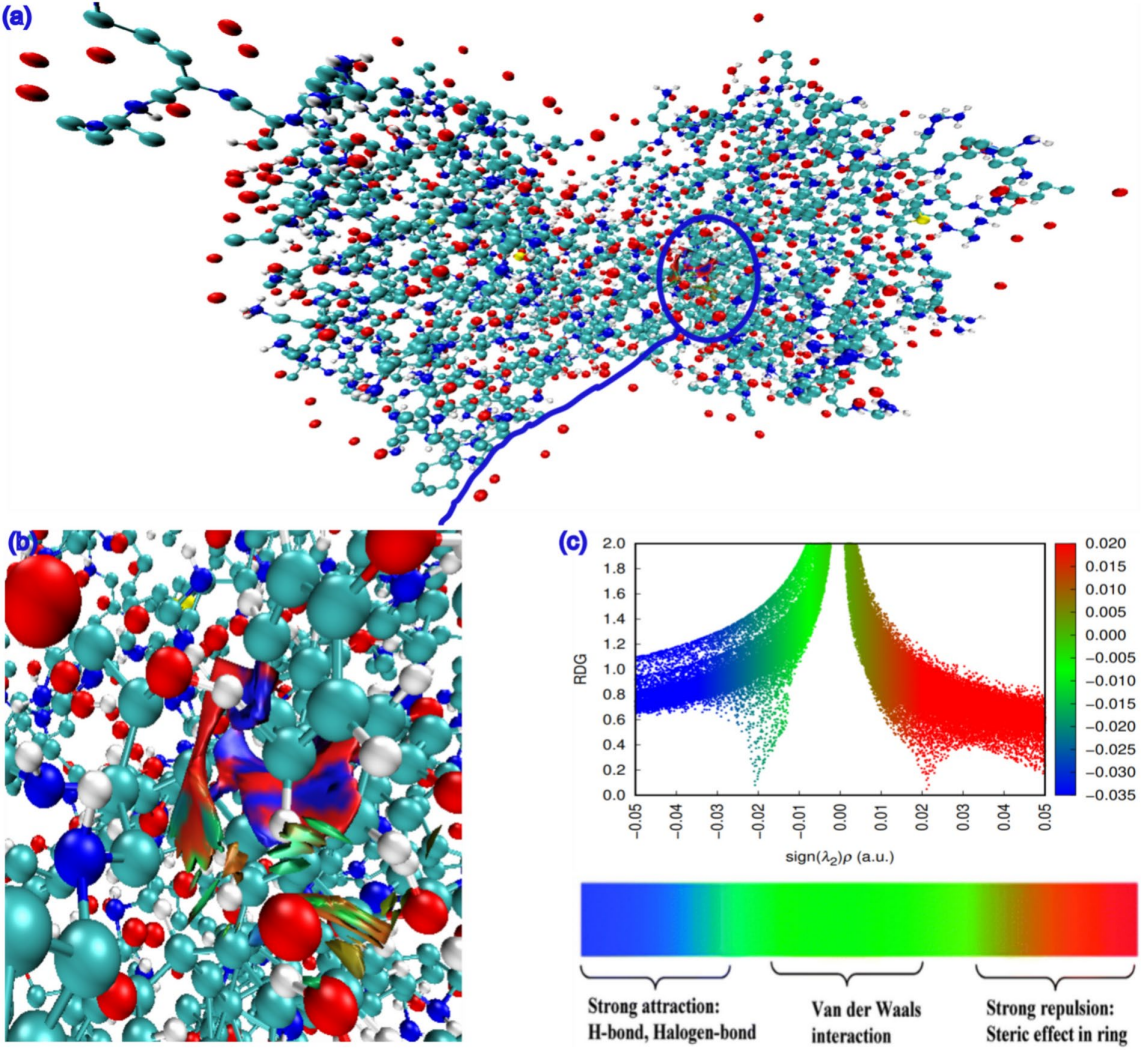
(**a**) top view and (**b**) zoomed view of the NCI isosurface, as well as (**c**) the RDG isosurface of the HPCR@1CGL protein compound adsorption.

After examining diverse HPCR reactivities, we ascertained distinct local reactivities that necessitated additional investigation into the molecule’s adsorption and interactions with many proteins, including (PDB: 3RJ3), (PDB: 7AXD), (PDB: 6DUK), and (PDB: 1CGL). We chose the 1CGL protein to investigate the interactions between it and the HPCR molecule. Non-covalent interactions (NCI) and reduced density gradient (RDG) are two theoretical approaches that we utilized to optimize the HPCR and 1CGL proteins. We did this by using the Materials Studio software’s Forcite features. The RDG and NCI isosurfaces were produced using the software packages Gnuplot and Multiwfn. Top and bottom views of the NCI isosurface and RDG isosurface of the adsorption of the HPCR compound onto the 1CGL protein are shown in (Fig. 13). When we examine these results, we see blue, green, and red colors, demonstrating the presence of strong attractions (H-bonds) and Van der Waals interactions^39,41,42^. These results demonstrate the local and global reactivity of the HPCR molecule and corroborate the experimental results.

## Conclusions

The study highlights the anti-proliferating activities of HPCR were studied. IC_50_ values were identified for cancer cell lines (U-87 MG, MCF-7, A549) and normal cell line (HDF) after treatment with HPCR and the Cisplatin as standard drug. The IC_50_ values were [71 ± 10 μM/ml (U-87), 92.3 ± 7.3 μM/ml (A549), 112.3 ± 5.4 μM/ml (MCF-7), and 146.7 ± 12 μM/ml (HDF), p ≤ 0.05]. A significant selective growth inhibitory activity against U-87 and A549 cell lines was obtained at an HPCR concentration of 100 μM. In addition, the results obtained from the Molecular Docking study, simulating their interactions, highlight that the utilization of HPCR within the active sites of all receptors is valuable not only for predictive purposes but also for optimizing its anticancer effects. The MOE docking module (version 2015) was utilized to assess the extent of inhibition for the HPCR compound against four cancer-related proteins (3RJ3, 7AXD, 6DUK, and 1CGL). The need for new alternatives in cancer treatment is driven by the limitations of existing therapies, the challenge of drug resistance, and the demand for more personalized and affordable options. Continued research and innovation, such as the development of compounds like C-4-hydroxyphenylcalix[4]resorcinarene, are essential to advancing cancer treatment and improving outcomes for patients worldwide.

## Data Availability

The data presented in this study are available upon request from the corresponding author.
